# Effects of Intensity of Facial Expressions on Amygdalar Activation Independently of Valence

**DOI:** 10.3389/fnhum.2016.00646

**Published:** 2016-12-20

**Authors:** Huiyan Lin, Miriam Mueller-Bardorff, Martin Mothes-Lasch, Christine Buff, Leonie Brinkmann, Wolfgang H. R. Miltner, Thomas Straube

**Affiliations:** ^1^Institute of Applied Psychology, Guangdong University of FinanceGuangzhou, China; ^2^Laboratory for Behavioral and Regional Finance, Guangdong University of Finance, GuangzhouGuangdong, China; ^3^Institute of Medical Psychology and Systems Neuroscience, University of MuensterMuenster, Germany; ^4^Department of Biological and Clinical Psychology, Friedrich-Schiller-University JenaJena, Germany

**Keywords:** facial expression, intensity, arousal, valence, amygdala, visual cortex, insula

## Abstract

For several stimulus categories (e.g., pictures, odors, and words), the arousal of both negative and positive stimuli has been shown to modulate amygdalar activation. In contrast, previous studies did not observe similar amygdalar effects in response to negative and positive facial expressions with varying intensity of facial expressions. Reasons for this discrepancy may be related to analytical strategies, experimental design and stimuli. Therefore, the present study aimed at re-investigating whether the intensity of facial expressions modulates amygdalar activation by circumventing limitations of previous research. Event-related functional magnetic resonance imaging was used to assess brain activation while participants observed a static neutral expression and positive (happy) and negative (angry) expressions of either high or low intensity from an ecologically valid, novel stimulus set. The ratings of arousal and intensity were highly correlated. We found that amygdalar activation followed a u-shaped activation pattern with highest activation to high intense facial expressions as compared to low intensity facial expressions and to the neutral expression irrespective of valence, suggesting a critical role of the amygdala in valence-independent arousal processing of facial expressions. Additionally, consistent with previous studies, intensity effects were also found in visual areas and generally increased activation to angry versus happy faces were found in visual cortex and insula, indicating enhanced visual representations of high arousing facial expressions and increased visual and somatosensory representations of threat.

## Introduction

From an evolutionary perspective, fast perception of emotional information is critical for human beings in forming rapid and appropriate behavioral responses to adapt to the environment. According to the dimensional model of emotion (e.g., Barrett, 1995, 1998), emotional information is generally perceived from two dimensions: valence (positive to negative) and arousal (high to low). How these two dimensions of emotional information are processed in the brain has become an important topic of research in psychology and human neuroscience (e.g., Davis and Whalen, 2001; Sabatinelli et al., 2005; Kuppens et al., 2013; Lang and Bradley, 2013; Sieger et al., 2015; Styliadis et al., 2015).

An important brain region in this field of research is the amygdala. Classically, amygdalar activation was supposed to be associated with negative emotional valence, particularly threat (e.g., Davis, 1992; LaBar et al., 1998; Barad et al., 2006). However, there is increasing evidence that for several categories of emotional stimuli (e.g., pictures, odors, or words), amygdalar activation is modulated by stimulus arousal independently of valence. In terms of emotional pictures, amygdalar activation was found to be stronger for both positive and negative as compared to neutral pictures (e.g., Kensinger and Schacter, 2006; Kanske et al., 2011). Pictures with high arousal were found to yield enhanced activations in the amygdala, regardless of emotional valence (e.g., Phan et al., 2003; Sabatinelli et al., 2005; Fastenrath et al., 2014; Bonnet et al., 2015). For odors, Royet et al. (2000) reported stronger amygdalar activation for pleasant and unpleasant as compared to neutral odors. High as compared to low arousing odors were shown to elicit larger activity in the amygdala for both pleasant and unpleasant odors (e.g., Anderson et al., 2003; Winston et al., 2005). With respect to words, both positive and negative as compared to neutral words were found to produce stronger activation in the amygdala (e.g., Hamann and Mao, 2002; Straube et al., 2011b; Laeger et al., 2012). Amygdalar activation was shown to enhance with the increasing arousal of emotional words (e.g., Lewis et al., 2007). Taken together, these findings suggest that the amygdalar activation is involved in the processing of arousal (Sabatinelli et al., 2005) and is associated with general emotional relevance of stimuli (Davis and Whalen, 2001; Sander et al., 2003; Phelps and LeDoux, 2005).

Remarkably, studies concerned with the processing of facial expressions showed an inconsistent picture with regard to effects of amygdalar activation. Studies that used negative (e.g., fearful or angry), positive and neutral facial expressions yielded mixed findings with regard to arousal effects. Some studies reported valence-specific effects, particularly threat-specific effects (e.g., Whalen et al., 1998; LeDoux, 2003; Gamer and Büchel, 2009; Inagaki et al., 2012; Furl et al., 2013; Sauer et al., 2014); whereas other studies showed modulations by emotion in general (Yang et al., 2002; Santos et al., 2011) or no emotional effects at all on amygdalar activation (e.g., Fitzgerald et al., 2006; Sato et al., 2010). These discrepancies may be related to multiple factors, such as attention (e.g., Pessoa et al., 2002; Straube et al., 2011a), face habituation (e.g., Breiter et al., 1996; Wright et al., 2001), ambiguity of facial expression (Adams et al., 2003), task condition (e.g., implicit or explicit; e.g., Critchley et al., 2000; Habel et al., 2007) and arousal differences between positive and negative expressions (e.g., Sauer et al., 2014). With regard to the last point, studies that vary arousal of facial expressions in a controlled way (i.e., using positive and negative expressions of matched arousal values) should be highly informative, similar to studies with other kinds of emotional stimuli mentioned above.

Previous studies that investigated amygdalar activation in response to facial expressions with varying expression intensity, which is highly correlated with arousal ratings, consistently failed to show valence-independent intensity effects on amygdalar activation. For example, a block design study by Morris et al. (1996) found that amygdalar activation increased with increasing intensity for fearful expressions, but decreased with increasing intensity for happy expressions. The findings clearly suggested a role for the amygdala in processing the potential threat-relevance of fearful expressions varying in intensity from the happiest to the most fearful. However, Gerber et al. (2008) reported that amygdalar activation is negatively correlated with the arousal for various facial expressions (i.e., scare, surprise, anger, disgust, happiness, excitement, neutral, sadness, sleepiness, boredom, and contentment). In contrast to these findings, N’Diaye et al. (2009) and Sarkheil et al. (2013) did not observe any significant intensity effects in amygdalar activation for fearful, angry, and happy facial expressions when dynamic facial expressions were used. Another block design study by Mattavelli et al. (2014) used fearful, angry, disgusted, sad, neutral and happy expressions and manipulated happy expressions in two intensities (100 and 25%), showing that threat-related expressions (e.g., fearful and angry) produced stronger activity in the amygdala than did neutral and 25% happy expressions, but no other effects were significant. Because not all negative expressions enhanced amygdalar activation than did neutral expressions and there was no intensity effect for happy expressions, the authors suggested that the amygdalar activation is associated with threat, but not valence or arousal, at least during face processing.

The discrepant findings of the above mentioned studies may be related to analytical strategies, experimental design and stimuli used in these studies. In terms of analysis, Morris et al. (1996) analyzed the intensity effects of fearful and happy expressions only within a regression model within a cluster detected by a main effect of fearful versus happy expressions. Thus, the analytical strategy was not apt to detect the presence of valence-independent intensity effects. For experimental design, Morris et al. (1996) and Mattavelli et al. (2014) adopted a block design, which is known to be prone to cause habituation, expectation, and regulation effects. These unwanted effects might have decreased the impact of intensity (see Breiter et al., 1996; Wright et al., 2001). Even though event-related design was used in some other studies (Gerber et al., 2008; N’Diaye et al., 2009); similar unwanted effects may appear when the presentation frequency was different between positive and negative facial expressions. With regard to stimuli, N’Diaye et al. (2009) and Sarkheil et al. (2013) used dynamic facial expressions, which were unfolded from neutral to some other expressions varying in valence and intensity. However, changes of facial expressions have been found to affect amygdalar activation (Harris et al., 2012, 2014). More importantly, facial expressions used in some previous studies (N’Diaye et al., 2009; Mattavelli et al., 2014) were not rated. However, happy as compared to angry or fearful expressions are more common in everyday life and therefore, are often perceived less arousing and intense. In this case, the relevance or significance or faces may be reduced, resulting in altering the effects of amygdalar activations (Somerville and Whalen, 2006; Straube et al., 2011a). Therefore, it is still unclear whether these studies (N’Diaye et al., 2009; Mattavelli et al., 2014) used sufficiently strong expressions of happiness or not, with comparable arousal ratings between happy and threat-related expressions.

The present study aimed at re-investigating whether intensity of facial expression modulates amygdalar activation and whether potential intensity effects depend on emotional valence of facial expressions. To address this issue, blood oxygenation level-dependent (BOLD) activation was assessed by means of event-related functional magnetic resonance imaging (fMRI) while participants were presented with static facial pictures. The faces showed a neutral expression and angry and happy expressions of either high or low intensity. For all faces normative valence and arousal ratings were available. The number of presentation was balanced for each experimental condition, especially regarding angry and happy faces. The faces were taken from an own newly developed face database which includes happy expressions with high arousal levels (Müller-Bardorff et al., 2016). All facial expressions were presented in randomized order to reduce any kinds of block-design-related habituation, expectation and regulation effects. We hypothesized that, by circumventing limitations of previous studies, the intensity of facial expressions would affect amygdalar activation regardless of valence of facial expressions.

## Materials and Methods

### Participants

Sixteen healthy participants (19–28 years, *M* = 22.56, *SD* = 2.78; 10 females) were recruited from the student population of the University of Jena, Germany. Participants were right-handed as determined by the Edinburgh Handedness Inventory (Oldfield, 1971) and had normal and corrected-to-normal vision. None of the participants reported a history of neurological or psychiatric illness. The experimental procedures were approved by the Ethics Committee of the University of Jena, and written informed consent was obtained from all participants prior to participation.

### Stimuli

Facial expressions were selected from Jena 3D Face Database (J3DFD), which is described in detail elsewhere (Müller-Bardorff et al., 2016). The J3DFD contains 608 pictures portraying 32 Caucasian models (16 females) showing happy, angry, fearful, sad, surprised, and disgusted facial expressions at three intensity levels plus neutral expressions. All models were part of an amateur acting group (20–27 years, *M* = 22.58 years, *SD* = 1.97, 1–10 years of acting experience). The models received written instructions prior to the photo session entailing (a) situations typically evoking the targeted emotions, (b) bodily sensations accompanying the targeted emotions, and (c) changes in the face associated with the targeted emotions (see Ekman, 2007 for more detail). For the realization of three distinct intensity levels, actors were firstly asked to express each emotion in a strong and clear way while recalling a critical situation evoking the emotion of interest. Thereafter, actors were asked to show a stronger intensity level than the preceding emotion expression (high intensity). Finally, actors were instructed to show the expression in a weaker but still a clear way (low intensity). For all facial pictures, eyes, noses, and mouths were located at similar positions. Outer parts of the neck and shoulders were removed.

**FIGURE 1:**
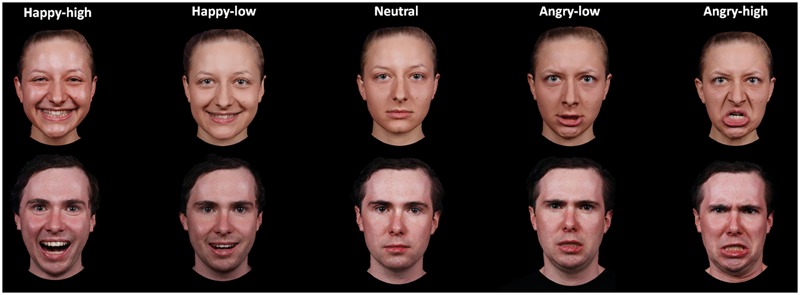
**Examples of facial expressions for all experimental conditions**.

A subset of 60 pictures was selected for the present study. These pictures portrayed 12 identities (six females), displaying high and low intensity angry and happy expressions plus a neutral expression. The selection was based on rating data on intensity, arousal and valence by an independent sample of 44 participants (29 females; 19–39 years, *M* = 23.07, *SD* = 4.28). Participants rated angry, happy and neutral expressions for arousal (ranging from 1 to 9; 1 = very low, 9 = very high) and valence (ranging from 1 to 9; 1 = very negative, 9 = very positive) and angry and happy facial expressions for intensity (ranging from 1 to 7; 1 = very low, 7 = very high). For analysis of intensity ratings, a repeated measure analysis of variance (ANOVA) with the factors expression valence (angry versus happy) and expression intensity (high versus low) was performed. In addition, other ANOVAs with the factor facial expression (happy-high, happy-low, neutral, angry-low and angry-high) were performed for arousal and valence ratings. Results on intensity ratings showed that there was a main effect of intensity [*F*(1,43) = 571.89, *p* < 0.001], with higher ratings for high compared to low intensity facial expressions. The interaction between valence and intensity was also significant [*F*(1,43) = 17.97, *p* < 0.001]. The intensity ratings were higher for high as compared to low facial expressions for both angry and happy faces, though to a different extent [happy: *F*(1,43) = 569.75, *p* < 0.001; angry: *F*(1,43) = 189.79, *p* < 0.001]. With respect to the arousal ratings, there was an effect of facial expression [*F*(2,99) = 90.54, *p* < 0.001; corrected by Greenhouse–Geisser]. *Post hoc* tests showed that both angry- and happy-high faces were rated as more arousing than were angry- and happy-low and neutral faces, and angry- and happy-low faces were rated as more arousing than were neutral faces (all *p* < 0.05; corrected by Bonferroni correction). For valence ratings, the effect of facial expression was also significant [*F*(2,81) = 310.80, *p* < 0.001; corrected by Greenhouse–Geisser]. Happy-high and -low facial expressions were rated as more positive compared to all other expressions, neutral facial expressions were rated as more positive than angry-high and -low faces, and angry-low faces were rated as more positive than angry-high faces (all *p* < 0.05; corrected by Bonferroni correction). For descriptive data, please refer to **Table 1** in more detail. The mean accuracy of emotional classification was at 0.86 (*SE* = 0.01). Examples of facial pictures are illustrated in **Figure 1**.

**Table 1:** Mean ratings of intensity, valence, attractiveness, and distinctiveness for each facial expression.

	Happy-high	Happy-low	Neutral	Angry-low	Angry-high
Intensity	5.51 (0.10)	3.69 (0.13)	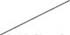	3.89 (0.10)	5.22 (0.11)
Arousal	5.30 (0.22)	3.79 (0.21)	2.20 (0.14)	4.35 (0.21)	5.45 (0.24)
Valence	6.69 (0.19)	6.53 (0.13)	5.29 (0.11)	3.18 (0.12)	2.45 (0.10)

*Standard errors (SE) are given in parentheses.*

### Procedure

Functional magnetic resonance imaging-scanning consisted of two runs. In each run, each facial picture mentioned in the *Stimuli* section was presented twice. Therefore, the experiment consisted of 240 trials (48 trials per condition × 5 conditions: happy-high, happy-low, neutral, angry-low, and angry-high). Each face picture was presented for 2000 ms and the mean interval between two facial pictures was 4240 ms. During presentations of facial pictures, participants were asked to judge the gender of the face so that participants paid attention to the face stimuli. The instructions for the gender judgment task emphasized speed as well as accuracy. Responses were given via one of two buttons of a response box with either the index or the middle finger of the right hand. The sequence of the pictures was randomized and the assignment of genders to buttons was counterbalanced across participants. All stimuli were presented against a dark background. Presentations of stimuli and recordings of behavioral responses were controlled by Presentation Software (Neurobehavioral Systems, Inc., Albany, CA, USA).

### Behavioral Data Recording and Analysis

Response accuracy and times of button presses for the facial pictures were recorded. Behavioral data were analyzed by means of repeated measure analysis of variance (ANOVA) with facial expression (happy-high, happy-low, neutral, angry-low, and angry-high) as within-subject factor using SPSS 22 software (SPSS, Inc., Chicago, IL, USA). In the analysis of response times, trials only with correct responses were included. Greenhouse–Geisser was applied to correct degrees of freedom and *p*-values of repeated measurements and Bonferroni correction was used to correct *p*-values of *post hoc* tests, if appropriate. A probability level of *p* < 0.05 was thought to be statistical significant. All data are expressed as M ± SE.

### fMRI Data Acquisition and Analysis

Scanning was performed in a 3-Tesla magnetic resonance scanner (TrioTim; Siemens, Erlangen, Germany). Two runs of 365 volumes each (35 axial slice per volume, thickness = 3 mm, 0.5 mm gap, in-plane resolution = 3 mm × 3 mm) were acquired for each participants using a T2^∗^-weighted echo-planar sequence (TE = 30 ms, flip angle = 90°, matrix = 96 × 96, field of view = 192 mm, TR = 2080 ms). For each run, the first 10 volumes were discarded to ensure steady-state tissue magnetization. In addition, a high-resolution T1-weighted anatomical volume was recorded.

Image preprocessing and analysis were performed using Brain Voyager QX (Brain Innovation, Maastricht, the Netherlands). The volumes were realigned to the first volume to minimize effects of head movements, and slice time correction was conducted. Further preprocessing comprised spatial (8 mm full-width half-maximum isotropic Gaussian kernel) and temporal (high-pass filter: 10 cycles per run; low pass filter: 2.8 s, linear trend removal) smoothing. The anatomical and functional images were co-registered and normalized to the Talairach space (Talairach and Tournoux, 1988).

Statistical analysis was performed by multiple linear regression of the signal time course at each voxel. The expected BOLD signal change for each event type (predictor) was modeled by a hemodynamic response function. Firstly, voxel-wise statistical maps were generated and predictor estimates were computed for each individual. The present study included five predictors: happy-high, happy-low, neutral, angry-low and angry-high. Then, a random-effects group analysis of individual contrasts of predictor estimates was performed. There were two approaches of analysis, the general approach and the approach taken normative arousal ratings into account. With respect to the general approach, analysis was conducted for three different contrasts (as well as their reversed counterparts). The first contrast modeled that high intense facial expressions showed increased activations compared to low intense facial expressions and to the neutral expression specified by a weighted contrast (balanced contrast values for happy-high, happy-low, neutral, angry-low and angry-high: 3, -2, -2, -2, 3; the reversed contrast: -3, 2, 2, 2, -3). The next contrast modeled valence (happy versus angry) effects independently of intensity (balanced contrast values for happy-high, happy-low, neutral, angry-low, and angry-high: 1, 1, 0, -1, -1 or -1, -1, 0, 1, 1). The last contrast modeled the interaction between expression intensity and valence (balanced contrast values for happy-high, happy-low, neutral, angry-low, and angry-high: 1, -1, 0, 1, -1 or -1, 1, 0, -1, 1). For the approach including normative ratings, normative mean arousal ratings for each facial expression (see **Table 1**) were used for contrast values, after linear transformation to generate a balanced sum contrast value = 0. This contrast in fact modeled a u-shaped function across the facial expression predictors (happy-high, happy-low, neutral, angry-low, and angry-high).

Analysis was conducted for the whole brain and the amygdala as region of interest (ROI), respectively. For the ROI analysis, amygdalar activation was analyzed in both hemispheres separately according to the Wake Forest University (WFU) – Pick Atlas (Maldjian et al., 2003). Statistical parametric maps that resulted from voxel-wise analysis were considered statistically significant for clusters that survived a correction for multiple comparisons. We used the approach as implemented in Brain Voyager (Goebel et al., 2006) on the basis of a 3D extension of the randomization procedure described by Forman et al. (1995). Voxel-level threshold was initially set to *p* < 0.005 (uncorrected). The correction criterion was based on the estimate of the maps’ spatial smoothness and on an iterative procedure (Monte Carlo stimulation) for estimating cluster-level false-positive rates. After 1000 iterations, the minimum cluster size threshold that yielded a cluster-level false-positive rate of 5% was applied to the statistical maps.

## Results

### Behavioral Results

For response accuracy and times, the ANOVAs did not show an effect of facial expression (*p* > 0.05). For descriptive data, please see **Table 2**.

**Table 2:** Mean accuracy (%) and response times (ms) for each facial expression.

	Happy-high	Happy-low	Neutral	Angry-low	Angry-high
Accuracy	97.45 (0.66)	98.18 (0.42)	97.27 (0.70)	97.92 (0.76)	97.14 (0.71)
Response times	713.14 (37.03)	700.35 (34.71)	712.93 (39.99)	703.87 (36.75)	719.51 (36.69)

*Standard errors (SE) are given in parentheses.*

### fMRI Results

#### ROI Analysis

##### The general approach

**FIGURE 2:**
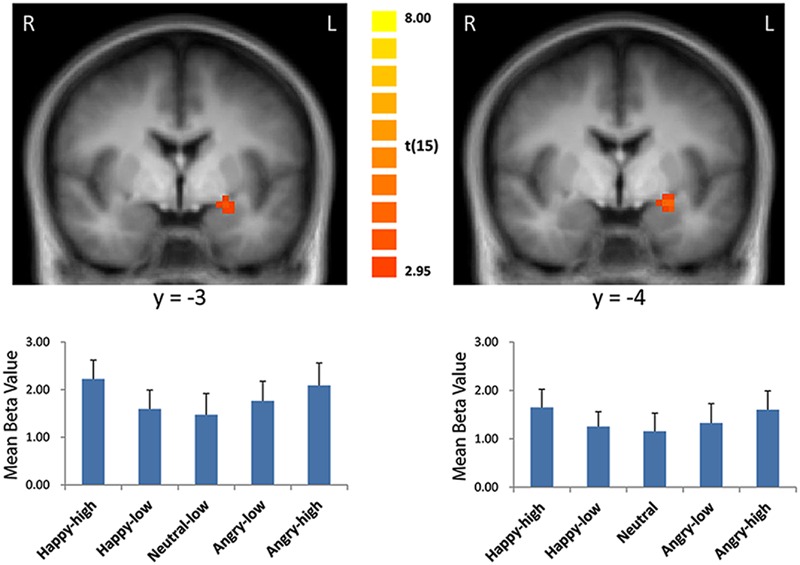
**Enhanced activation in the left amygdala to high intensity as compared to low intensity emotional facial expressions and to the neutral expression (left: with the general approach; right: with the approach using normative arousal ratings).** The plots display contrasts of parameter estimates (mean ± SE for cluster mean). Radiological convention: left = right.

For the contrast modeling increased activation to high as compared to low intensity expressions, increased activation was found in the left amygdala (*x* = -21, *y* = 0, *z* = -8; size = 329 mm^3^; *t* = 3.21, *p* < 0.05, corrected; see **Figure 2**). There were no significant results for the other contrasts (i.e., the reversed intensity contrast, the valence contrasts and the interaction contrasts; *p* > 0.05).

##### The approach using normative arousal ratings

Consistent with findings using the general approach, we found that activation in the amygdala followed a u-shaped function with activation depending on arousal levels (*x* = -21, *y* = 0, *z* = -9; size = 675 mm^3^; *t* = 3.79, *p* < 0.05, corrected; see **Figure 2**).

#### Whole Brain Analysis

##### The general approach

**FIGURE 3:**
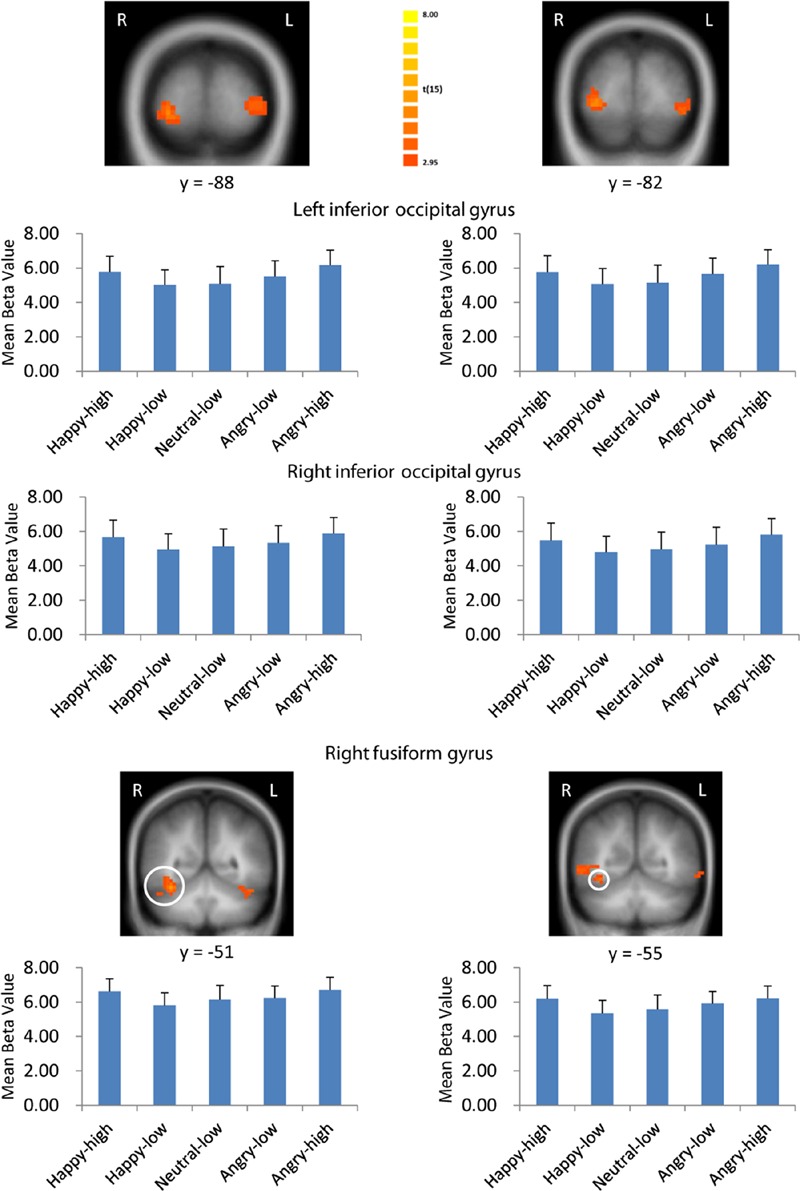
**Visual activation to high intensity as compared to low intensity emotional facial expressions and to the neutral expression.** Larger activation was found with both the general approach (left column) and the approach based on normative arousal ratings (right column) in inferior occipital gyrus and fusiform gyrus. The plots display contrasts of parameter estimates (mean ± SE for local cluster peaks). Radiological convention: left = right.

There were several areas showing intensity effects (**Table 3**). High compared to low intensity expressions elicited stronger activation in bilateral occipital gyrus and fusiform gyrus (see **Figure 3** for visual activation) and left post-central gyrus. In contrast, activation in right medial frontal gyrus and left inferior and superior frontal gyrus and middle temporal gyrus was increased in response to low as compared to high intensity expressions (all *p* < 0.05, corrected).

**Table 3:** Significant activations for high intensity facial expressions as compared to low intensity facial expressions and to the neutral expression.

Region of activation	Hemisphere	Peak x	Peak y	Peak z	Cluster size (mm^3^)	*t*
**High > Low + Neutral**						
Fusiform gyrus	R	38	-55	-11	4998	3.44
Inferior occipital gyrus	R	30	-84	-1	4164	3.64
Post-central gyrus	L	-59	-28	36	406	3.21
Inferior occipital gyrus and fusiform gyrus	L	-40	-73	-6	11346	3.41
**Low + Neutral > High**						
Medial frontal gyrus	R	11	57	12	326	3.21
	R	14	41	26	787	3.45
Inferior frontal gyrus	L	-33	32	13	444	3.43
Middle temporal gyrus	L	-59	7	-8	281	3.54

We also detected valence effects as summarized in **Table 4**. Angry compared to happy expressions elicited larger activation in bilateral fusiform gyrus, in right inferior, middle and superior frontal gyrus, inferior parietal lobule and middle occipital gyrus and in left insula, middle temporal gyrus, posterior cingulate, inferior occipital gyrus and cerebellum. No brain regions were more strongly activated for happy as compared to angry expressions (all *p* < 0.05, corrected).

**Table 4:** Significant activations for valence contrasts.

Region of activation	Hemisphere	Peak x	Peak y	Peak z	Cluster size (mm^3^)	*t*
**Angry > Happy**						
Inferior frontal gyrus	R	48	32	13	632	3.39
Middle frontal gyrus	R	45	18	26	283	3.33
Superior frontal gyrus	R	12	7	63	393	3.63
Inferior parietal lobule	R	40	-36	38	270	3.20
Fusiform gyrus	R	40	-72	-14	292	3.08
Middle occipital gyrus	R	37	-78	6	1485	3.28
Insula	L	-38	17	-2	395	3.29
Fusiform gyrus	L	-41	-39	-10	860	3.71
Middle temporal gyrus	L	-45	-54	6	479	3.27
Posterior cingulate	L	-25	-64	13	544	3.35
Inferior occipital gyrus	L	-38	-76	-2	879	3.17
Cerebellum	L	-38	-50	-29	898	3.43
**Happy > Angry**						
No brain regions were activated						

Intensity effects that interact with valence are shown in **Table 5**. An interaction of valence and intensity was found in bilateral insula, in right cingulate gyrus, precentral gyrus, post-central gyrus, superior temporal gyrus, posterior cingulate and cerebellum and in left anterior cingulate, middle frontal gyrus and inferior parietal lobule (all *p* < 0.05, corrected).

**Table 5:** Significant activations for valence-by-intensity contrasts.

Region of activation	Hemisphere	Peak x	Peak y	Peak z	Cluster size (mm^3^)	*t*
**Happy-high + Low-angry > Happy-low + Angry-high**					
Cingulate gyrus	R	13	12	25	375	3.42
	R	25	-22	41	2536	3.47
Precentral gyrus	R	57	-2	21	551	3.28
	R	55	-11	40	378	3.25
Insula	R	42	-20	12	4224	3.53
Post-central gyrus	R	42	-28	54	906	3.22
Superior temporal gyrus	R	40	-40	9	611	3.37
	R	29	-51	35	2507	3.50
Posterior cingulate	R	13	-67	12	277	3.14
Cerebellum	R	30	-49	-26	422	3.32
	R	10	-74	-20	598	3.23
Anterior cingulate	L	-17	16	-6	282	3.18
	L	-22	-26	39	1256	3.41
Middle frontal gyrus	L	-48	15	31	2944	3.37
Insula	L	-29	-5	1	862	3.23
	L	-46	-21	19	6204	3.35
Inferior parietal lobule	L	-33	-45	45	836	3.41
**Happy-low + Angry-high > Happy-high + Low-angry**					
No brain regions were activated						

##### The approach using normative arousal ratings

Consistent with findings using the general approach, we found that activation in the same visual areas followed more or less a u-shaped function with activation depending on arousal levels (*p* < 0.05, corrected; see **Table 6**; **Figure 3**). Additionally, we found also significant effects in right superior temporal gyrus and left temporal lobe (**Table 6**).

**Table 6:** Significant activations for the u-shaped function based on normative arousal ratings.

Region of activation	Hemisphere	Peak x	Peak y	Peak z	Cluster size (mm^3^)	*t*
Superior temporal gyrus	R	47	-30	5	675	3.48
Inferior occipital gyrus and fusiform gyrus	R	38	-66	-2	6885	3.45
Temporal lobe	L	-40	34	-4	567	3.51
Fusiform gyrus	L	-41	-37	-14	324	3.11
Inferior occipital gyrus	L	-44	-71	-4	4752	3.47
	L	-21	-93	-2	270	3.20

*Activation in visual areas was partially part of extended clusters across regions. The table shows in this case results separated for anatomical regions.*

## Discussion

In the present study, we investigated whether the intensity of facial expressions is associated with increase of amygdalar activation independently of valence. We found increased activation in the amygdala to high intensity facial expressions as compared to low intensity and neutral facial expressions regardless of valence. Furthermore, additional analysis revealed that amygdalar activation followed a u-shaped function across valence categories following normative arousal ratings. This suggests that the amygdala plays a critical role in processing emotional relevance of facial expressions irrespectively of valence. In addition, whole brain analysis revealed arousal-driven activations mainly in visual areas (e.g., inferior occipital gyrus and fusiform gyrus) and threat-driven activation mainly in visual areas and insula.

The findings of the present study differ in several points from those of previous studies which investigated amygdalar activation to facial expressions with varying intensity. Morris et al. (1996) reported an intensity effect of facial expression on amygdalar activation depending on valence, and other studies showed enhanced amygdalar activation for low arousal facial expressions (Gerber et al., 2008) or did not observe significant effects of the intensity (N’Diaye et al., 2009; Sarkheil et al., 2013; Mattavelli et al., 2014). As mentioned in the introduction the discrepancies may be related to analytical strategies, experimental design and stimuli. The regression model as implemented by Morris et al. (1996) was not appropriate to detect valence-independent intensity effects. Furthermore, effects of presentation frequency between high and low arousal facial expressions may have influenced results too. Furthermore, block design studies are known to cause problems related to habituation, expectation and regulation (Breiter et al., 1996; Wright et al., 2001). Dynamic facial expressions need additional resources to process dynamic information (Harris et al., 2012, 2014). Moreover, the intensity and arousal levels of happy expressions may be lower than those of threat-related expressions in several studies (N’Diaye et al., 2009; Mattavelli et al., 2014), which may reduce the relevance and significance of faces and the effects of amygdalar activations as a result (Somerville and Whalen, 2006; Straube et al., 2011a). In the present study, we presented differential facial expressions in randomized order to limit block design-related problems. Static facial expressions were used, with controlled presentation duration of the whole blow facial expressions. Emotional faces, whose arousal and intensity had been assessed in a pilot study, were adopted where the arousal of happy-high and happy-low faces was similar to that of angry-high and angry-low faces, respectively. We also used only one negative expression to provide a balanced frequency of positive and negative faces.

Our results suggest that amygdalar activation is at least partially driven by arousal of facial expressions, which is strongly correlated with the intensity of facial expression. The findings of the present study are also in accordance with previous studies with respect to other stimulus categories that showed that high as compared to low arousing emotional stimuli produced stronger amygdalar activation independent of stimulus valence (e.g., pictures, odors, and words; Hamann and Mao, 2002; Anderson et al., 2003; Sabatinelli et al., 2005; Winston et al., 2005; Straube et al., 2011b; Laeger et al., 2012; Bonnet et al., 2015). Taken these studies and our present study together, it can be concluded that the amygdala is a detector of subjective relevance of stimuli across different categories of stimuli, including faces.

In general, our findings on amygdalar activation are relevant for the on-going debate about whether valence, arousal, or other aspects of emotional stimuli elicit amygdalar activation. Some authors propose that amygdalar activation may be relevant to vigilance, significance, or general relevance of stimuli (Davis and Whalen, 2001; Sander et al., 2003; Phelps and LeDoux, 2005). While a number of studies supported the hypothesis of valence-specific activation, valence effects were suggested to result from lower arousal levels for positive as compared to negative stimuli (Somerville and Whalen, 2006; Straube et al., 2008). When the arousal of positive stimuli is not high enough, arousal effects for positive stimuli may be reduced or even abolished. However, this seems not to be the case for highly arousing positive stimuli (e.g., Yang et al., 2002). Therefore, the selection of highly arousing positive stimuli may be an important factor to detect arousing effects for happy faces in the amygdala.

Furthermore, while previous studies did not find effects of expression intensity on amygdalar activation across valence categories (Morris et al., 1996; Gerber et al., 2008; N’Diaye et al., 2009; Sarkheil et al., 2013; Mattavelli et al., 2014), due to reasons discussed above; there is a large number of studies which indicated that amygdalar activation is associated with responses to relevant and salient facial information (e.g., averted gaze; Fitzgerald et al., 2006; Straube et al., 2010; Santos et al., 2011). Analogously, our present findings are in line with accounts that posit a role for the amygdala in coding salience of stimuli.

In addition to the amygdala, we found increased activation in visual cortex (e.g., fusiform gyrus and inferior occipital gyrus) for high intensity facial expressions as compared to low intensity facial expressions and the neutral expression regardless of valence, which is in line with previous studies (N’Diaye et al., 2009; Sarkheil et al., 2013). According to Haxby et al.’s (2000) model, fusiform gyrus and inferior occipital gyrus are parts of a face processing network that is involved in early perception of facial features and the representation of facial identity. Based on this model, the increased activation for high intensity facial expressions in the present study may be related to enhanced sensory representations of highly arousing facial expressions (e.g., Kanwisher et al., 1997; Vuilleumier et al., 2001). Furthermore, previous studies have suggested that the amygdala has extensive connections with many cortical/subcortical areas, such as back-projections to visual areas that may modulate sensory processing based on emotional signals (e.g., Pascual-Leone and Walsh, 2001; Vuilleumier et al., 2004). Accordingly, in the present study, the intensity effect on activations in fusiform gyrus and inferior occipital gyrus may be in relevance to back-projections from amygdala.

It must be noted that we detected also valence specific activations. These comprised specifically increased activations to angry compared to happy expressions in visual cortex and insula. The findings are consistent with previous studies, which showed that visual cortex and insula are activated more strongly in response to negative stimuli (e.g., Geday et al., 2003; Cunningham et al., 2004; Straube et al., 2011b; Furl et al., 2013). As stated in the above-mentioned paragraph, visual cortex plays a role in early perception of facial features and the representation of identity (Haxby et al., 2000). Additionally, the insula is involved in integrating the awareness of emotion through internal bodily state (i.e., interception; Craig, 2009). Accordingly, the findings in the present study may imply that angry as compared to happy expressions are associated with enhanced visual representations and representations of bodily states.

We would like to mention some limitations of our study and suggest outlines for future research. Firstly, while we now found intensity-independent amygdalar activations, this effect was obtained based on a small sample size. Future studies may expand the sample size to further investigate this issue. Secondly, we used normative ratings to model effects; future studies might use ratings of participants from the actual experiment. Thirdly, the present study investigated only one kind of negative facial expression. It remains unclear whether effects are comparable when other negative expressions (e.g., threat-unrelated negative expressions) are used (Mattavelli et al., 2014). Furthermore, while the present study found intensity effects on amygdalar activation independently of valence, amygdalar activation generally depends on attentional conditions (Pessoa et al., 2002; Straube et al., 2011a). It has been suggested that the amygdala might show a threat-specific activation under attentional load (Straube et al., 2008). Whether this threat advantage under high attentional load, which might be related to evolutionary adaptation, modulates intensity effects in amygdala activation remains to be investigated in future studies. Additionally, the amygdala consists of several subnuclei with different functions and activation profiles (e.g., Amorapanth et al., 2000; Hoffman et al., 2007; LeDoux, 2007). For instance, the basolateral amygdala (including the lateral, the basal and the accessory basal nuclei) of the amygdala is supposed to form the main visual input region of the amygdala and to be involved in the encoding of facial expression (e.g., Gamer et al., 2010; Boll et al., 2011; Sauer et al., 2014); whereas the corticomedial amygdala, which comprises of the medial, cortical and central nuclei, is linked to vigilance, attention and the integration of contextual information (e.g., Gamer et al., 2010; Boll et al., 2011; Sauer et al., 2014). Future studies should increase spatial resolution to investigate effects of intensity of facial expression on amygdalar activation in more detail. Moreover, fMRI studies have repeatedly shown differences between females as compared to males in response to emotional stimuli (Cahill, 2006; Domes et al., 2010; Whittle et al., 2011; Kret and De Gelder, 2012). Surprisingly, to the best of our knowledge, it seems that no studies have investigated the modulation of gender of faces and participants on the interaction intensity by valence. In future studies, researchers may consider the factors gender of face stimuli and gender of participants to investigate the related issues.

## Conclusion

The present study provides evidence that high as compared to low intensity and to the neutral facial expressions lead to enhanced amygdalar activation regardless of emotional valence, and in follows a u-shaped function depending on arousal ratings. Our findings, therefore, support the hypothesis that amygdalar activation is associated with arousal independently of valence with no exception for facial expressions. In line with previous studies, our findings showed enhanced visual cortex activation for high intensity facial expressions and increased visual cortex and insular activation for angry facial expressions, suggesting enhanced visual representations of high arousing facial expressions and enhanced visual representations and representations of bodily states for angry expressions.

## Author Contributions

HL was involved in data analysis and manuscript drafting and revises. MM-B was involved in data analysis and manuscript revises. CB and LB made the figures and revised the manuscript. MM-L was involved in study design, execution, data analysis, and manuscript revises. WM and TS were involved in study design and manuscript revises. We have read and approved the manuscript and agree to be accountable for all aspects of the work in ensuring that questions related to the accuracy or integrity of any part of the work are appropriately investigated and resolved.

## Conflict of Interest Statement

The authors declare that the research was conducted in the absence of any commercial or financial relationships that could be construed as a potential conflict of interest.

## References

[B1] Adams R. B. Gordon H. L. Baird A. A. Ambady N. Kleck R. E. (2003). Effects of gaze on amygdala sensitivity to anger and fear faces. *Science* 300 1536–1536. 10.1126/science.1082244 12791983

[B2] Amorapanth P. LeDoux J. E. Nader K. (2000). Different lateral amygdala outputs mediate reactions and actions elicited by a fear-arousing stimulus. *Nat. Neurosci.* 3 74–79. 10.1038/71145 10607398

[B3] Anderson A. K. Christoff K. Stappen I. Panitz D. Ghahremani D. G. Glover G. (2003). Dissociated neural representations of intensity and valence in human olfaction. *Nat. Neurosci.* 6 196–202. 10.1038/nn1001 12536208

[B4] Barad M. Gean P. W. Lutz B. (2006). The role of the amygdala in the extinction of conditioned fear. *Biol. Psychiat.* 60 322–328. 10.1016/j.biopsych.2006.05.029 16919522

[B5] Barrett L. F. (1995). Valence focus and arousal focus: individual differences in the structure of affective experience. *J. Pers. Soc. Psychol.* 69 153–166. 10.1037/0022-3514.69.1.153

[B6] Barrett L. F. (1998). Discrete emotions or dimensions? The role of valence focus and arousal focus. *Cogn. Emot.* 12 579–599. 10.1080/026999398379574

[B7] Boll S. Gamer M. Kalisch R. Büchel C. (2011). Processing of facial expressions and their significance for the observer in subregions of the human amygdala. *Neuroimage* 56 299–306. 10.1016/j.neuroimage.2011.02.021 21320610

[B8] Bonnet L. Comte A. Tatu L. Millot J. L. Moulin T. Medeiros de Bustos E. (2015). The role of the amygdala in the perception of positive emotions: an “intensity detector”. *Front. Behav. Neurosci.* 9:178 10.3389/fnbeh.2015.00178 PMC4493392 26217205

[B9] Breiter H. C. Etcoff N. L. Whalen P. J. Kennedy W. A. Rauch S. L. Buckner R. L. (1996). Response and habituation of the human amygdala during visual processing of facial expression. *Neuron* 17 875–887. 10.1016/S0896-6273(00)80219-6 8938120

[B10] Cahill L. (2006). Why sex matters for neuroscience. *Nat. Rev. Neurosci.* 7 477–484. 10.1038/nrn1909 16688123

[B11] Craig A. D. (2009). How do you feel–now? The anterior insula and human awareness. *Nat. Rev. Neurosci.* 10 59–70. 10.1038/nrn2555 19096369

[B12] Critchley H. Daly E. Phillips M. Brammer M. Bullmore E. Williams S. (2000). Explicit and implicit neural mechanisms for processing of social information from facial expressions: a functional magnetic resonance imaging study. *Hum. Brain Mapp.* 9 93–105. 10680766 10.1002/(SICI)1097-0193(200002)9:2<93::AID-HBM4>3.0.CO;2-Z PMC6872127

[B13] Cunningham W. A. Raye C. L. Johnson M. K. (2004). Implicit and explicit evaluation: fMRI correlates of valence, emotional intensity, and control in the processing of attitudes. *J. Cogn. Neurosci.* 16 1717–1729. 10.1162/0898929042947919 15701224

[B14] Davis M. (1992). The role of the amygdala in fear and anxiety. *Ann. Rev. Neurosci.* 15 353–375. 10.1146/annurev.ne.15.030192.002033 1575447

[B15] Davis M. Whalen P. J. (2001). The amygdala: vigilance and emotion. *Mol. Psychiat.* 6 13–34. 10.1038/sj.mp.4000812 11244481

[B16] Domes G. Schulze L. Böttger M. Grossmann A. Hauenstein K. Wirtz P. H. (2010). The neural correlates of sex differences in emotional reactivity and emotion regulation. *Hum. Brain Mapp.* 31 758–769. 10.1002/hbm.20903 19957268 PMC6871188

[B17] Ekman P. (2007). “The directed facial action task,” in *Handbook of Emotion Elicitation and Assessment* eds Coan J. A. Allen J. J. B. (New York, NY: Oxford University Press) 47–53.

[B18] Fastenrath M. Coynel D. Spalek K. Milnik A. Gschwind L. Roozendaal B. (2014). Dynamic modulation of amygdala–hippocampal connectivity by emotional arousal. *J. Neurosci.* 34 13935–13947. 10.1523/JNEUROSCI.0786-14.2014 25319690 PMC6705297

[B19] Fitzgerald D. A. Angstadt M. Jelsone L. M. Nathan P. J. Phan K. L. (2006). Beyond threat: amygdala reactivity across multiple expressions of facial affect. *Neuroimage* 30 1441–1448. 10.1016/j.neuroimage.2005.11.003 16368249

[B20] Forman S. D. Cohen J. D. Fitzgerald M. Eddy W. F. Mintun M. A. Noll D. C. (1995). Improved assessment of significant activation in functional magnetic resonance imaging (fMRI): use of a cluster-size threshold. *Magn. Reson. Med.* 33 636–647. 10.1002/mrm.1910330508 7596267

[B21] Furl N. Henson R. N. Friston K. J. Calder A. J. (2013). Top-down control of visual responses to fear by the amygdala. *J. Neurosci.* 33 17435–17443. 10.1523/JNEUROSCI.2992-13.2013 24174677 PMC6618361

[B22] Gamer M. Büchel C. (2009). Amygdala activation predicts gaze toward fearful eyes. *J. Neurosci.* 29 9123–9126. 10.1523/JNEUROSCI.1883-09.2009 19605649 PMC6665435

[B23] Gamer M. Zurowski B. Büchel C. (2010). Different amygdala subregions mediate valence-related and attentional effects of oxytocin in humans. *Proc. Natl. Acad. Sci. U.S.A.* 107 9400–9405. 10.1073/pnas.1000985107 20421469 PMC2889107

[B24] Geday J. Gjedde A. Boldsen A. S. Kupers R. (2003). Emotional valence modulates activity in the posterior fusiform gyrus and inferior medial prefrontal cortex in social perception. *Neuroimage* 18 675–684. 10.1016/S1053-8119(02)00038-1 12667845

[B25] Gerber A. J. Posner J. Gorman D. Colibazzi T. Yu S. Wang Z. (2008). An affective circumplex model of neural systems subserving valence, arousal, and cognitive overlay during the appraisal of emotional faces. *Neuropsychologia* 46 2129–2139. 10.1016/j.neuropsychologia.2008.02.032 18440572 PMC2486369

[B26] Goebel R. Esposito F. Formisano E. (2006). Analysis of functional image analysis contest (FIAC) data with brainvoyager QX: From single-subject to cortically aligned group general linear model analysis and self-organizing group independent component analysis. *Hum. Brain Mapp.* 27 392–401. 10.1002/hbm.20249 16596654 PMC6871277

[B27] Habel U. Windischberger C. Derntl B. Robinson S. Kryspin-Exner I. Gur R. C. (2007). Amygdala activation and facial expressions: explicit emotion discrimination versus implicit emotion processing. *Neuropsychologia* 45 2369–2377. 10.1016/j.neuropsychologia.2007.01.023 17408704

[B28] Hamann S. Mao H. (2002). Positive and negative emotional verbal stimuli elicit activity in the left amygdala. *Neuroreport* 13 15–19. 10.1097/00001756-200201210-00008 11924878

[B29] Harris R. J. Young A. W. Andrews T. J. (2012). Morphing between expressions dissociates continuous from categorical representations of facial expression in the human brain. *Proc. Natl. Acad. Sci. U.S.A.* 109 21164–21169. 10.1073/pnas.1212207110 23213218 PMC3529057

[B30] Harris R. J. Young A. W. Andrews T. J. (2014). Dynamic stimuli demonstrate a categorical representation of facial expression in the amygdala. *Neuropsychologia* 56 47–52. 10.1016/j.neuropsychologia.2014.01.005 24447769 PMC3988993

[B31] Haxby J. V. Hoffman E. A. Gobbini M. I. (2000). The distributed human neural system for face perception. *Trends Cogn. Sci.* 4 223–233. 10.1016/S1364-6613(00)01482-0 10827445

[B32] Hoffman K. L. Gothard K. M. Schmid M. C. Logothetis N. K. (2007). Facial-expression and gaze-selective responses in the monkey amygdala. *Curr. Biol.* 17 766–772. 10.1016/j.cub.2007.03.040 17412586

[B33] Inagaki T. K. Muscatell K. A. Irwin M. R. Cole S. W. Eisenberger N. I. (2012). Inflammation selectively enhances amygdala activity to socially threatening images. *Neuroimage* 59 3222–3226. 10.1016/j.neuroimage.2011.10.090 22079507 PMC3348143

[B34] Kanske P. Heissler J. Schönfelder S. Bongers A. Wessa M. (2011). How to Regulate Emotion? Neural networks for reappraisal and distraction. *Cereb. Cortex* 21 1379–1388. 10.1093/cercor/bhq216 21041200

[B35] Kanwisher N. McDermott J. Chun M. M. (1997). The fusiform face area: a module in human extrastriate cortex specialized for face perception. *J. Neurosci.* 17 4302–4311. 9151747 10.1523/JNEUROSCI.17-11-04302.1997 PMC6573547

[B36] Kensinger E. A. Schacter D. L. (2006). Processing emotional pictures and words: effects of valence and arousal. *Cogn. Affect. Behav. Neurosci.* 6 110–126. 10.3758/CABN.6.2.110 17007232

[B37] Kret M. E. De Gelder B. (2012). A review on sex differences in processing emotional signals. *Neuropsychologia* 50 1211–1221. 10.1016/j.neuropsychologia.2011.12.022 22245006

[B38] Kuppens P. Tuerlinckx F. Russell J. A. Barrett L. F. (2013). The relation between valence and arousal in subjective experience. *Psychol. Bull.* 139 917–940. 10.1037/a0030811 23231533

[B39] LaBar K. S. Gatenby J. C. Gore J. C. LeDoux J. E. Phelps E. A. (1998). Human amygdala activation during conditioned fear acquisition and extinction: a mixed-trial fMRI study. *Neuron* 20 937–945. 10.1016/S0896-6273(00)80475-4 9620698

[B40] Laeger I. Dobel C. Dannlowski U. Kugel H. Grotegerd D. Kissler J. (2012). Amygdala responsiveness to emotional words is modulated by subclinical anxiety and depression. *Behav. Brain Res.* 233 508–516. 10.1016/j.bbr.2012.05.036 22659393

[B41] Lang P. J. Bradley M. M. (2013). Appetitive and defensive motivation: goal-directed or goal-determined. *Emot. Rev.* 5 230–234. 10.1177/1754073913477511 24077330 PMC3784012

[B42] LeDoux J. (2003). The emotional brain, fear, and the amygdala. *Cell. Mol. Neurobiol.* 23 727–738. 10.1023/A:1025048802629 14514027 PMC11530156

[B43] LeDoux J. (2007). The amygdala. *Curr. Biol.* 17 868–874. 10.1016/j.cub.2007.08.005 17956742

[B44] Lewis P. A. Critchley H. D. Rotshtein P. Dolan R. J. (2007). Neural correlates of processing valence and arousal in affective words. *Cereb. Cortex* 17 742–748. 10.1093/cercor/bhk024 16699082 PMC2267931

[B45] Maldjian J. A. Laurienti P. J. Kraft R. A. Burdette J. H. (2003). An automated method for neuroanatomic and cytoarchitectonic atlas-based interrogation of fMRI data sets. *Neuroimage* 19 1233–1239. 10.1016/S1053-8119(03)00169-1 12880848

[B46] Mattavelli G. Sormaz M. Flack T. Asghar A. U. Fan S. Frey J. (2014). Neural responses to facial expressions support the role of the amygdala in processing threat. *Soc. Cogn. Affect. Neurosci.* 9 1684–1689. 10.1093/scan/nst162 24097376 PMC4221207

[B47] Morris J. S. Frith C. D. Perrett D. I. Rowland D. Young A. W. Calder A. J. (1996). A differential neural response in the human amygdala to fearful and happy facial expressions. *Nature* 383 812–815. 10.1038/383812a0 8893004

[B48] Müller-Bardorff M. Schulz C. Peterburs J. Bruchmann M. Mothes-Lasch M. Miltner W. (2016). Effects of emotional intensity under perceptual load: an event-related potentials (ERPs) study. *Biol. Psychol.* 117 141–149. 10.1016/j.biopsycho.2016.03.006 26995785

[B49] N’Diaye K. Sander D. Vuilleumier P. (2009). Self-relevance processing in the human amygdala: gaze direction, facial expression, and emotion intensity. *Emotion* 9 798–806. 10.1037/a0017845 20001123

[B50] Oldfield R. C. (1971). The assessment and analysis of handedness: the Edinburgh inventory. *Neuropsychology* 9 97–113. 10.1016/0028-3932(71)90067-4 5146491

[B51] Pascual-Leone A. Walsh V. (2001). Fast backprojections from the motion to the primary visual area necessary for visual awareness. *Science* 292 510–512. 10.1126/science.1057099 11313497

[B52] Pessoa L. McKenna M. Gutierrez E. Ungerleider L. G. (2002). Neural processing of emotional faces requires attention. *Proc. Natl. Acad. Sci. U.S.A.* 99 11458–11463. 10.1073/pnas.172403899 12177449 PMC123278

[B53] Phan K. L. Taylor S. F. Welsh R. C. Decker L. R. Noll D. C. Nichols T. E. (2003). Activation of the medial prefrontal cortex and extended amygdala by individual ratings of emotional arousal: a fMRI study. *Biol. Psychiat.* 53 211–215. 10.1016/S0006-3223(02)01485-3 12559653

[B54] Phelps E. A. LeDoux J. E. (2005). Contributions of the amygdala to emotion processing: from animal models to human behavior. *Neuron* 48 175–187. 10.1016/j.neuron.2005.09.025 16242399

[B55] Royet J. P. Zald D. Versace R. Costes N. Lavenne F. Koenig O. (2000). Emotional responses to pleasant and unpleasant olfactory, visual, and auditory stimuli: a positron emission tomography study. *J. Neurosci.* 20 7752–7759. 11027238 10.1523/JNEUROSCI.20-20-07752.2000 PMC6772882

[B56] Sabatinelli D. Bradley M. M. Fitzsimmons J. R. Lang P. J. (2005). Parallel amygdala and inferotemporal activation reflect emotional intensity and fear relevance. *Neuroimage* 24 1265–1270. 10.1016/j.neuroimage.2004.12.015 15670706

[B57] Sander D. Grafman J. Zalla T. (2003). The human amygdala: an evolved system for relevance detection. *Rev. Neurosci.* 14 303–316. 10.1515/REVNEURO.2003.14.4.303 14640318

[B58] Santos A. Mier D. Kirsch P. Meyer-Lindenberg A. (2011). Evidence for a general face salience signal in human amygdala. *Neuroimage* 54 3111–3116. 10.1016/j.neuroimage.2010.11.024 21081170

[B59] Sarkheil P. Goebel R. Schneider F. Mathiak K. (2013). Emotion unfolded by motion: a role for parietal lobe in decoding dynamic facial expressions. *Soc. Cogn. Affect. Neurosci.* 8 950–957. 10.1093/scan/nss092 22962061 PMC3831559

[B60] Sato W. Kochiyama T. Uono S. Yoshikawa S. (2010). Amygdala integrates emotional expression and gaze direction in response to dynamic facial expressions. *Neuroimage* 50 1658–1665. 10.1016/j.neuroimage.2010.01.049 20096793

[B61] Sauer A. Mothes-Lasch M. Miltner W. H. Straube T. (2014). Effects of gaze direction, head orientation and valence of facial expression on amygdala activity. *Soc. Cogn. Affect. Neurosci.* 9 1246–1252. 10.1093/scan/nst100 23946006 PMC4127025

[B62] Sieger T. Serranová T. Růžička F. Vostatek P. Wild J. Š’astná D. (2015). Distinct populations of neurons respond to emotional valence and arousal in the human subthalamic nucleus. *Proc. Natl. Acad. Sci. U.S.A.* 112 3116–3121. 10.1073/pnas.1410709112 25713375 PMC4364224

[B63] Somerville L. H. Whalen P. J. (2006). Prior experience as a stimulus category confound: an example using facial expressions of emotion. *Soc. Cogn. Affect. Neurosci.* 1 271–274. 10.1093/scan/nsl040 18985113 PMC2555423

[B64] Straube T. Langohr B. Schmidt S. Mentzel H. J. Miltner W. H. (2010). Increased amygdala activation to averted versus direct gaze in humans is independent of valence of facial expression. *Neuroimage* 49 2680–2686. 10.1016/j.neuroimage.2009.10.074 19883773

[B65] Straube T. Mothes-Lasch M. Miltner W. H. (2011a). Neural mechanisms of automatic processing of emotional information from faces and voices. *Br. J. Psychol.* 102 830–848. 10.1111/j.2044-8295.2011.02056.x 21988387

[B66] Straube T. Pohlack S. Mentzel H. J. Miltner W. H. (2008). Differential amygdala activation to negative and positive emotional pictures during an indirect task. *Behav. Brain Res.* 191 285–288. 10.1016/j.bbr.2008.03.040 18466987

[B67] Straube T. Sauer A. Miltner W. H. (2011b). Brain activation during direct and indirect processing of positive and negative words. *Behav. Brain Res.* 222 66–72. 10.1016/j.bbr.2011.03.037 21440008

[B68] Styliadis C. Loannides A. A. Bamidis P. D. Papadelis C. (2015). Distinct cerebellar lobules process arousal, valence and their interaction in parallel following a temporal hierarchy. *Neuroimage* 110 149–161. 10.1016/j.neuroimage.2015.02.006 25665964

[B69] Talairach J. Tournoux P. (1988). *Co-Planar stereotaxic atlas of the human brain: 3-dimensional proportional system: an approach to cerebral imaging*. Stuttgart: Thieme.

[B70] Vuilleumier P. Armony J. L. Driver J. Dolan R. J. (2001). Effects of attention and emotion on face processing in the human brain: an event-related fMRI study. *Neuron* 30 829–841. 10.1016/S0896-6273(01)00328-2 11430815

[B71] Vuilleumier P. Richardson M. P. Armony J. L. Driver J. Dolan R. J. (2004). Distant influences of amygdala lesion on visual cortical activation during emotional face processing. *Nat. Neurosci.* 7 1271–1278. 10.1038/nn1341 15494727

[B72] Whalen P. J. Rauch S. L. Etcoff N. L. McInerney S. C. Lee M. B. Jenike M. A. (1998). Masked presentations of emotional facial expressions modulate amygdala activity without explicit knowledge. *J. Neurosci.* 18 411–418. 9412517 10.1523/JNEUROSCI.18-01-00411.1998 PMC6793390

[B73] Whittle S. Yücel M. Yap M. B. Allen N. B. (2011). Sex differences in the neural correlates of emotion: evidence from neuroimaging. *Biol. Psychol.* 87 319–333. 10.1016/j.biopsycho.2011.05.003 21600956

[B74] Winston J. S. Gottfried J. A. Kilner J. M. Dolan R. J. (2005). Integrated neural representations of odor intensity and affective valence in human amygdala. *J. Neurosci.* 25 8903–8907. 10.1523/JNEUROSCI.1569-05.2005 16192380 PMC6725588

[B75] Wright C. I. Fischer H. Whalen P. J. McInerney S. C. Shin L. M. Rauch S. L. (2001). Differential prefrontal cortex and amygdala habituation to repeatedly presented emotional stimuli. *Neuroreport* 12 379–383. 10.1097/00001756-200102120-00039 11209954

[B76] Yang T. T. Menon V. Eliez S. Blasey C. White C. D. Reid A. J. (2002). Amygdalar activation associated with positive and negative facial expressions. *Neuroreport* 13 1737–1741. 10.1097/00001756-200210070-00009 12395114

